# LAYPERSON’S UNDERSTANDING OF GEROTRANSCENDENCE

**DOI:** 10.1093/geroni/igae098.3537

**Published:** 2024-12-31

**Authors:** Oscar Ribeiro, Taiane Abreu, Laetitia Teixeira, Lia Araujo

**Affiliations:** University of Aveiro, Aveiro, Aveiro, Portugal; University of Aveiro, Aveiro, Aveiro, Portugal; University of Porto, Porto, Porto, Portugal; Polytechnic Institute of Viseu, Viseu, Viseu, Portugal

## Abstract

Gerotranscendence is an adaptive theory of aging that posits a mindset shift across three dimensions: cosmic, coherence, and solitude. Despite its consolidation in recent years, the theory’s depth continues to evolve, stimulating a need for broader perspectives to enhance its practical comprehension. This study aimed to explore laypersons’ perceptions regarding gerotranscendence’s aspects. Three focus groups were conducted with a total of 18 participants, ranging in age from 58 to 98 years old (mean=79.5). Open-ended questions were used to collect information regarding what gerotranscendence and its dimensions meant to them; thoughts on the components of the theory after a brief explanation were also obtained. Transcripts from recorded sessions underwent content analysis and organized under two overarching themes: general assumptions and personal perceptions. The latter theme encompassed two sub-themes highlighting participants’ identification of personal experiences, as well as their diverse and complementary interpretations of the theory. Initially, participants encountered challenges grasping the theory’s terminology. Subsequent clarification facilitated their recognition and resonance with gerotranscendence’s components. Further discussions exposed associations between concepts of gerotranscendence and other theoretical frameworks of successful aging, notably the life cycle theory and socio-emotional selectivity theory. This interconnection highlights the theory’s relevance within broader aging frameworks. Engaging lay individuals in gerotranscendence discussions yields profound insights into both its fundamental principles and culturally embedded nuances. By associating theoretical concepts with lived experiences, such dialogues enrich the understanding of aging paradigms, paving the way for more holistic approaches to aging research and practice.

